# Experimental demonstration of magnetoplasmon polariton at InSb(InAs)/dielectric interface for terahertz sensor application

**DOI:** 10.1038/s41598-017-13394-0

**Published:** 2017-10-13

**Authors:** Jan Chochol, Kamil Postava, Michael Čada, Jaromír Pištora

**Affiliations:** 1 VSB – Technical University of Ostrava, Nanotechnology Centre, 17. listopadu 15/2172, 708 33 Ostrava, Poruba Czech Republic; 2 Dalhousie University, Department of Electrical and Computer Engineering, 6299 South St, Halifax, NS B3H 4R2 Canada; 3 VSB – Technical University of Ostrava, Department of Physics, 17. listopadu 15/2172, 708 33 Ostrava, Poruba Czech Republic

## Abstract

We experimentally demonstrate surface plasmon resonance (SPR) in the terahertz range in InSb and InAs. The surface plasmon is excited on the interface between a thin polymer film and the semiconductor using a silicon prism in Otto configuration. The low effective mass of InSb and InAs permits tuning of the SPR by an external magnetic field in the transversal configuration. The data show a good agreement with a model. Strong excitation of the surface plasmon is present in both materials, with a shifting of resonance position by more than 100 GHz for the field of 0.25 T, to both higher and lower energies with opposite orientation of the magnetic field. Applicability of the terahertz SPR sensor is discussed, along with modeled design for the Kretschmann configuration.

## Introduction

The surface plasmon resonance (SPR)^1^, i.e. coupling of an electromagnetic wave to a collective oscillation of free carriers on the interface between a conductor and dielectric as a surface wave allows highly accurate sensing. This sensitivity to the properties and changes in the dielectric (analyte) has found its use primarily in biomedical applications^2^.

The spectral range in which SPR is utilized is dictated by the materials used. The prerequisite being a negative permittivity of the conductor. This role is suitably filled by noble metals in the visible and near infrared range. Noble metals, however, lose their SPR suitability for lower energies. The permittivity becomes highly negative with strong absorptions, permitting only weakly bound surface waves (Zenneck regime). Pushing the functionality of SPR devices towards the infrared and terahertz range would be useful in many applications. Namely the terahertz range (0.2–10 THz) is home to many interesting phenomena. Organic molecules, aqueous solutions, and many other compounds have distinguishable spectra in the THz range, which together with non-ionizing properties of the terahertz radiation permits its broad use in biomedicine and *in-vivo* characterization^3^. Absorption spectra from rotational transitions of gaseous analytes can be detected and analyzed through THz spectroscopy^4^. Moreover, THz spectroscopy has become indispensable in basic science and materials research, from dielectrics to superconductors^5^. The use of surface plasmon resonance has the potential to improve all of these areas. The natural high confinement of surface plasmons is valuable for investigation of samples with sub-wavelength sizes when in THz range the wavelengths are on the order hundreds of micrometers.

There is an ongoing research exploring other materials more applicable to lower frequencies SPR, with the potential candidates of conducting oxides^6^, graphene^7,8^, corrugated metal^9^, and semiconductors^10^.

The issue of surface plasmons and magnetoplasmons (plasmons, which behavior is influenced by an external magnetic field through magneto-optical effects)^11^ on semiconductors, namely InSb, has been investigated by the the research group of Palik, Hartstein, Burtstein, Wallis and Brion^12–16^ as the continuation of research of semiconductor properties. The experiments have however been limited to n-doped samples and measurements in the far-infrared range due to the absence of THz sources and detectors at the time of their research. The advent of modern usable terahertz technology meant that surface (magneto) plasmons on semiconductors in the THz have been investigated for their properties^17,18^ and applications, ranging from the THz radiation detection^19^ to nonreciprocal transmission and tunable lenses^20^. However, further advances in the design of prism-coupled SPR THz sensor remained in the theoretical domain^7,8,10,21^.

In this paper, we experimentally confirm and describe a THz SPR sensor based on InSb and InAs with the additional functionality of strong magnetic tuning of the surface plasmon resonance. The surface plasmon is coupled to an interface between the analyte (polymer film) and InSb/InAs via Si prism.

The section Theory describes the properties and constants of the materials used, including the influence of external magnetic field. The Experimental Demonstration section presents, in our knowledge, the first experimental demonstration of surface plasmon resonance in THz spectral range and presents the measured data, first without magnetic field tuning, then with magnetic tuning. The Discussion compiles our findings on the performance and sensitivity of the experimental setup and presents a design of a different configuration applicable for the THz sensor. Section Methods provides the details on the time-domain measurement, model, and data of permittivity of semiconductors, and modeling method used.

## Theory

**Figure 1:**
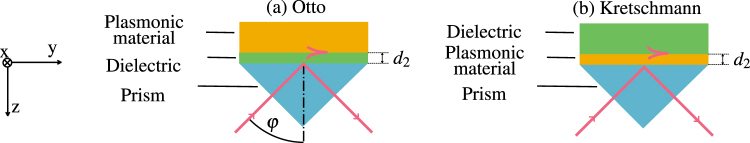
The coordinate system used throughout this work and a diagram of prism-coupled SPR solutions. The Otto configuration uses a bulk plasmonic material separated from the prism by a thin layer of a dielectric analyte. In the Kretschmann configuration, the plasmonic material is deposited as a thin film on the prism, with the dielectric analyte on top. In both cases, the prism is used to match the wavevector of the incident light to the wavevector of the surface plasmon polariton at the interface between the dielectric analyte and plasmonic material.

This section deals with the modeling and description of the optical parameters of the semiconductors. The permittivity of the semiconductors in the far-infrared and Terahertz domain is described using the Drude-Lorentz model

1 $${\varepsilon }_{r}={\varepsilon }_{\infty }-\mathop{\underbrace{\frac{{\omega }_{p}^{2}}{{\omega }^{2}+i{\gamma }_{p}\omega }}}\limits_{{\varepsilon }_{D}}+\mathop{\underbrace{\frac{{A}_{L}{\omega }_{L}^{2}}{{\omega }_{L}^{2}-{\omega }^{2}-i{\gamma }_{L}\omega }}}\limits_{{\varepsilon }_{L}}\mathrm{\ ,}$$

where the constant term *ε*_∞_ describes the background permittivity (high-frequency absorptions), the Drude term *ε*_*D*_ describes the contribution of free carriers and the Lorentz term *ε*_*L*_ comes from the lattice vibrations. In the Drude term, the plasma frequency is defined as

2 $${\omega }_{p}={(\frac{N{e}^{2}}{{\varepsilon }_{0}{m}^{\ast }})}^{\frac{1}{2}},$$

where *N* is the carrier concentration, *e* is the electron charge, *ε*_0_ is the permittivity of free space, *m* ^*^ = *m*_eff_ *m*_0_ is the effective mass of the charge carriers (*m*_0_ is the mass of electron in vacuum) and 1/*γ*_*p*_ = *τ*_*p*_ is the scattering time. The plasma frequency divided by $\sqrt{{\varepsilon }_{\infty }}$ is the frequency where the real part of permittivity crosses zero. The Lorentz term is characterized by the frequency *ω*_*L*_, the scattering time *τ*_*L*_ = 1/*γ*_*L*_, and the amplitude *A*_*L*_. If an external magnetic field is applied to the sample, it induces anisotropy in the Drude term. The typically used configurations are polar, longitudinal and transversal, where the magnetic field is in the *z*, *y* and *x* direction respectively. The coordinate system is shown in the Fig. 1.

We apply the magnetic field in the transversal configuration, sometimes called the Voight magnetoplasmonic configuration^11^, where the anisotropy affects only the TM polarization, leading to the strongest modulation of the surface plasmon polariton. The permittivity tensor is then

3 $${\hat{\varepsilon }}_{r}=[\begin{array}{ccc}{\varepsilon }_{xx} & 0 & 0\\ 0 & {\varepsilon }_{yy} & {\varepsilon }_{yz}\\ 0 & {\varepsilon }_{zy} & {\varepsilon }_{zz}\end{array}].$$

**Figure 2:**
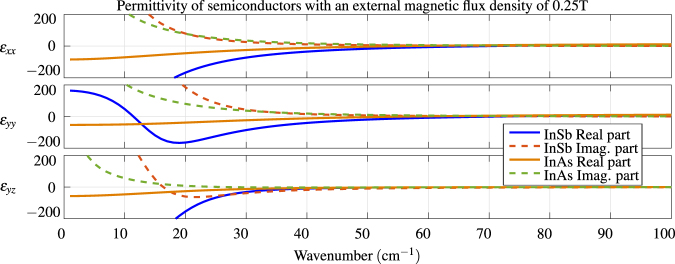
Tensor components of the permittivity of InSb and InAs in an external magnetic flux density of 0.25 T, calculated using Eq. (1,4,5) and parameters in Table 1. Without the magnetic field, the semiconductors are isotropic with values of *ε*_*xx*_ on the diagonal.

The *ε*_*xx*_ component stays the same as *ε*_*r*_ in (1) and *yy*, *zz*, *yz*, *zy* components change to

4 $${\varepsilon }_{yy}={\varepsilon }_{zz}={\varepsilon }_{\infty }-\frac{{\omega }_{p}^{2}({\omega }^{2}+i{\gamma }_{p}\omega )}{{({\omega }^{2}+i{\gamma }_{p}\omega )}^{2}-{\omega }_{c}^{2}{\omega }^{2}}+{\varepsilon }_{L},$$

5 $${\varepsilon }_{yz}=-{\varepsilon }_{zy}=-i\frac{{\omega }_{p}^{2}{\omega }_{c}\omega }{{({\omega }^{2}+i{\gamma }_{p}\omega )}^{2}-{\omega }_{c}^{2}{\omega }^{2}},$$

where *ω*_*c*_ is the cyclotron frequency, defined as

6 $${\omega }_{c}=\frac{e{B}_{x}}{{m}^{\ast }},$$

where *B*_*x*_ is the magnetic flux density in the *x* direction. The cyclotron frequency is inversely proportional to the effective mass of the carriers, which means that the magnetic modulation is also inversely proportional to the effective mass for the same applied flux density. The parameters of InSb and InAs are listed in the Table 1. Data for InSb are taken from^17^ and data for InAs from^22^. The permittivities of both InSb and InAs are plotted in Fig. 2.

**Table 1:** Permittivity parameters of InSb^17^ and InAs^22^.

	*ω*_*p*_/$\sqrt{{\varepsilon }_{\infty }}$ (cm^−1^)	*τ*_*p*_ 10^−13^ (s)	*ω*_*L*_ (cm^−1^)	*τ*_*L*_ 10^−12^ (s)	*A*_*L*_	*ε*_∞_	*m*_*eff*_
InSb	76.4	5.81	179.4	2.00	1.93	15.14	0.0169
InAs	67.4	2.12	217.7	3.63	2.82	14.02	0.0260

The reflectivity and field profiles for the treatment of the experimental data and modeling are calculated using the Berreman 4 × 4 matrix method^23^, allowing for an arbitrary anisotropy calculation. The dielectric is assumed under negligible dispersion, while plasmonic semiconductor dispersion is applied according to Eq.(1,4,5) and Table 1. When fitting the model to the data, the thickness and the refractive index of the dielectric were taken as the fitting parameters as well as the cyclotron frequency for measurements with an external magnetic field. The data were weighted with a low error in the resonance and larger error elsewhere, to get the accurate picture of the surface plasmon resonance.

## Experimental Demonstration of Surface Plasmon Resonance in Otto configuration

The typical configuration of an SPR sensor is based on the Kretschmann or Otto architecture, both depicted in Fig. 1. The high refractive index prism is used to match the wavevector of the evanescent wave to the surface plasmon polariton at the interface between the conductor and analyte. In the Kretschmann configuration, the conductor is deposited as a thin film on the prism and the analyte is placed on the top. In the Otto configuration, the conductive material is pressed towards the prism, with the dielectric-analyte sandwiched between them. While the former method is used in practice, we’ve opted for the Otto configuration, which utilizes a bulk conductor. The experimental configuration and setup are discussed in Section Methods. A sensor geometry in the Kretschmann configuration is analyzed in the Discussion section.

### Surface Plasmon Resonance using different semiconductors

Thick wafers (500 *μ*m) of InSb and InAs and a thick layer of Au on glass were used as the plasmonic materials. The dielectric was chosen as a thin polymer film (high-density polyethylene), about 15 *μ*m thick. They were pressed together using a manual anvil on a Silicon prism (*n* = 3.4164)^24^ with the angle of incidence of *ϕ* = 35°, originally an attenuated total reflection (ATR) system. The reference was the signal from the empty prism.

**Figure 3:**
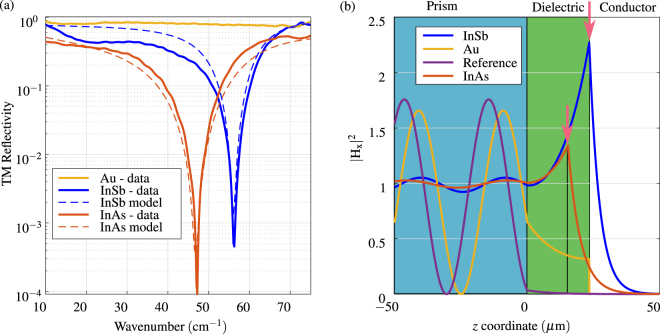
(**a**) Measured TM reflectivity of Otto configuration with different materials and data from a fitted model. The surface plasmon resonance is observed in InSb and InAs samples as sharp decrease in reflected intensity. (**b**) Calculated field profiles using the fitted model with highlighted field enhancement. The thickness of the dielectric for InAs is smaller due to different pressing strength of the manual anvil. The field profiles for semiconductors are calculated at the wavelengths of the resonances. For Au and reference, the wavelength was chosen as the same as for resonance in InSb.

The experimental data showing the prism coupled surface plasmon resonance are presented in Fig. 3. The left plot in Fig. 3(a) shows the transversal magnetic (TM, or p-polarized) reflectivity obtained from the experiment. The sharp decrease in reflectivity for both the InSb and InAs shows the surface plasmon resonance. On the other hand, Gold acts only as a reflector. A model was fitted to the measured data, denoted by dashed lines in the plot. Fitted values of the refractive index and the thickness of the dielectric are *n*_2_ = 1.625 and *d*_2_ = 23.4 *μ*m (with InSb) and *d*_2_ = 15.8 *μ*m (with InAs), the difference in thickness caused by the strength of the manual pressing. For fitting in InAs case, a fourth parameter was needed, the plasma frequency (with only a slight correction) to accurately model the resonance. Reasonable agreement between the data and the model were achieved. Due to the high refractive index of the Silicon prism, the angle of incidence is above the critical angle (*φ*_*c*_ = 28.4°) and the electromagnetic wave entering the dielectric is evanescent. Using the fitted model, it is possible to calculate the field profiles at the resonance. For Au and reference, the wavelength was chosen as the same as for resonance in InSb. The field profiles, normalized to the mean amplitude of the wave in the prism, depicted in Fig. 3(b) show a clear field concentration at the interface of the dielectric and InSb(InAs). The existence of surface plasmon resonance in clearly demonstrated by a strong and sharp increase of the electromagnetic field at the interface between the dielectric and the semiconductors, marked by arrows in Fig. 3(b). In contrast, the empty prism provides only evanescent wave penetration and Gold has only skin depth wave confinement, without any field concentration on the interface.

### Tuning of Surface Plasmon Resonance by an external magnetic field

**Figure 4:**
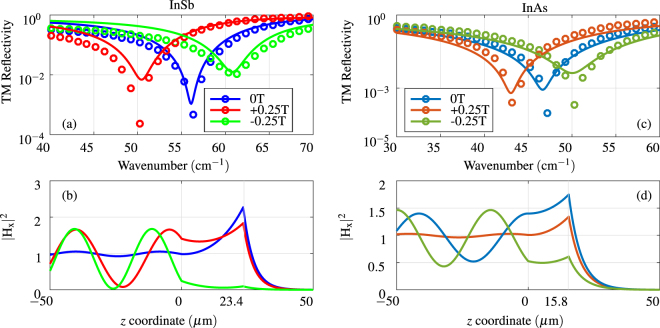
(**a**) Measurement and model of SPR on InSb at various external magnetic field in the transversal direction. (**b**) Calculated field profiles at respective wavelengths of the InSb SPR for each case, normalized to mean values in the prism. (**c**) Measurement and model of SPR on InAs at various external magnetic field in the transversal direction. (**d**) Calculated field profiles at respective wavelengths of the InAs SPR for each case, normalized to mean values in the prism.

**Figure 5:**
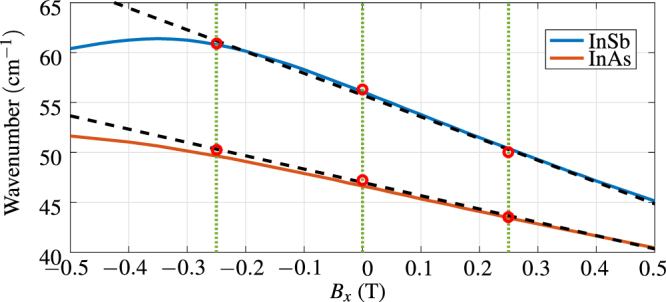
Influence of the magnetic field. The solid lines are modeled positions of the resonance as a function of transversal magnetic field. The red circles are the measured data points. The dashed lines are linear regressions based on the measured data. For flux densities stronger than ±0.25 T the dominant modulation comes quadratic dependence of diagonal components Eq. (4).

Another important property of THz InSb (InAs) plasmonics emerges with the application of external magnetic field. It has been demonstrated^17,22^, that the free carrier properties of InSb and InAs can be easily tuned with magnetic field. The transversal magneto-optical configuration, where the magnetic field is along the x-axis, i.e. perpendicular to the plane of incidence, changes the diagonal (*ε*_*yy*_, *ε*_*zz*_) and off-diagonal (*ε*_*yz*_ and *ε*_*zy*_) components of the permittivity tensor. The diagonal components are responsible for the plasmonic behavior while the off-diagonal components, which arise with external magnetic field are responsible for a non-symmetrical (nonreciprocal) response with the change of the orientation of the magnetic field. How the permittivity components change with respect to the magnetic field is described in Section Theory. The modulation in the permittivity of the conductor elicits a change in the observed surface plasmon resonance. Figure 4 show such change in the observed spectra with the application of the magnetic flux density *B*_*x*_ = ±0.25 T by small permanent magnets. Both InSb and InAs exhibit strong shift in the frequency position of the plasmonic resonance. The field profiles, calculated at the respective frequencies and normalized to the mean value in the prism, highlight the shift in SPR coupling. The shift, linearly proportional to the magnetic field for smaller flux densities (<0.25 T), is higher in InSb (160 GHz to each side) than for InAs (100 GHz) due to lower effective mass of carrier of InSb. Figure 5 shows the dependence of the spectral position of the resonance to applied magnetic field in the transversal direction for both InSb and InAs, in the measured configuration (angle of incidence o 35°, *n*_2_ = 1.625 and *d*_2_ = 23.4 *μ*m (with InSb) and *d*_2_ = 15.8 *μ*m for model with InAs). As per Eq. (4,5), there is linear dependence of magnetic field for off-diagonal components of permittivity tensor, which is valid for smaller fields. For larger fields, the quadratic dependence of diagonal components becomes dominant.

The use of magnetic tuning of the surface plasmon resonance broadens the possible application of semiconductor-based SPR THz sensor. First of all, the magnetic field can be used to fine-tune the surface plasmon resonance. By changing the properties of the semiconductor with a magnetic field we are able to find the strongest coupling of SPR for selected thickness and refractive index of the dielectric. This also means that we can scan the frequency based on our available magnetic field range and get a complete data set of SPR coupling of different strengths and frequency positions. Moreover, using a modulated magnetic field would also permit the use of a synchronous detection and a lock-in system, where the magneto-optical modulation would serve as a carrier wave. This setup would be beneficial in distinguishing weak signals from noise.

## Discussion of application of demonstrated SPR resonance for THz sensors

**Figure 6:**
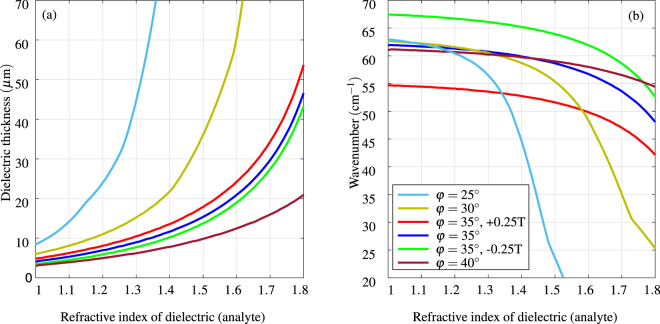
The calculated position of InSb SPR (TM reflectivity minimum) for different settings of the Otto configuration as a function of the refractive of the dielectric (analyte). For each refractive index and the angle of incidence, the thickness of the dielectric and resonance wavenumber are obtained simultaneously. For increasing angle of incidence (increasing propagation constant) the optimum is found for thinner dielectric layer (subplot (**a**)) and sensitivity to higher refractive indices (subplot (**b**)). For example, the highest sensitivity for analyte with refractive index 1.3–1.4 is with the angle of incidence of 25° and the resonances would be found in the range 45–55 cm^−1^. Blue, red and green curves correspond to values of our measurement setup.

The position and the strength of the surface plasmon resonance are intrinsically linked to the refractive index and thickness of the analyte as well as the complex permittivity of the conductor and the angle of incidence in the prism. Figure 6 shows the position of the strongest surface plasmon resonance based on the analyte refractive index for several cases of the angle of incidence and magnetic field in InSb. The data for Fig. 6 are calculated in the following way. For each configuration setting (angle of incidence and transversal magnetic field) the model is swept over three parameters: wavelength, refractive index of analyte and thickness of the analyte. The position of the strongest resonance (TM reflectivity minimum) is recorded and plotted. Note that the same effect as with varying the angle of incidence can be obtained by change of the refractive index of prism, because the tangential component of the prism propagation constant is *ν*_*y*_ = *n*_*prism*_ ⋅ sin *φ*, *n*_Si,prism_ = 3.4164. Assuming fixed thickness, the magnetic tuning offers excitation of SPR over range of frequencies. While the strength of the resonance varies, the excitation remains possible as shown in Fig. 4. Changing the angle of incidence or the material of the prism can be used to tune the sensitivity to desired range of refractive index and thickness of the analyte. For example, having the angle of incidence of 25° provides much higher sensitivity to analyte with the refractive index around 1.3 than configuration with the 35° angle of incidence. The actual configuration should be tailored to the need of a sensor.

### Theoretical Kretschmann configuration

**Figure 7:**
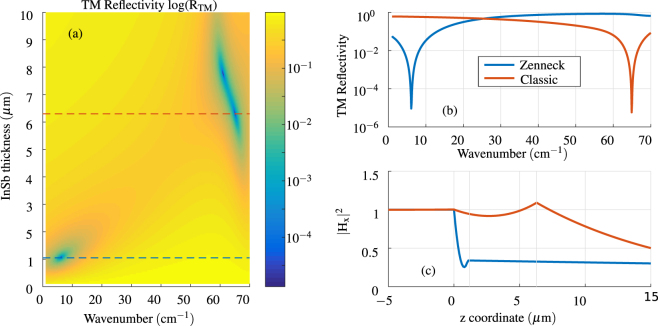
(**a**) Logarithmic contour of TM reflectivity simulation in the Kretschmann configuration as a function of InSb thickness and wavenumber. Refractive index of the dielectric (analyte) is 1.625. (**b**) TM reflectivity in Kretschmann configurations for two different InSb thicknesses 1.05 *μ*m and 6.3 *μ*m, indicated by the dashed lines in subplot (**a**). (**c**) Field profile of resonances showed in subplot (**b**), normalized to mean value in the prism.

While we have used the Otto configuration (Fig. 1(a)) for experimental simplicity, it is also possible to excite THz SPR in the Kretschmann configuration (Fig. 1(b)). This could offer more commercially viable solution, similar to visible light SPR sensors or Terahertz ATR modules, which utilize a fixed prism along with necessary focusing optics. However, it requires the thickness of semiconductor in the range of several *μ*m. Figure 7 shows models of such case, with bulk of dielectric (n = 1.625) on top of layer of InSb on a Silicon prism with the angle of incidence of 35 degrees. The TM Reflectivity sweep of InSb thickness and wavelength shows an interesting property. Two distinct minima are observed, corresponding to plasmonic resonances. Their reflectivities are shown top right with field profiles on the bottom right of Fig. 7. First, a 1.05 μm thick InSb hold a surface plasmon at 6.2 cm^−1^ (186 GHz, 1.61 mm) in Zenneck regime, typical for longer wavelength. The dispersion of this type of surface plasmon is very close to the dispersion of light in the dielectric analyte. The majority of the energy is carried in the dielectric, with very little confinement in the conductor, as it is shown in the field profile. On the other hand, with thickness 6.3 *μ*m, the configurations holds a classical surface plasmon resonance at 65 cm^−1^ (1.95 THz, 153.8 *μ*m), with confinement at the interface between both media. This shows that Kretschmann configuration is also well applicable for THz SPR sensor. The same principles, which were shown for Otto configuration - tuning by magnetic field and changing sensitivity for different refractive index of the analyte, are also present here.

## Conclusion

We have demonstrated experimental feasibility of exciting surface plasmon resonance at the interface of InSb(InAs)/dielectric in the Terahertz range using a high refractive index prism, previously described only theoretically. The measured data agree with the model. The model predicts the possibility to tune sensitivity to different dielectric materials. The low-band-gap semiconductors used, InSb and InAs, combine plasmonic behavior with gigantic magneto-optics response in THz range, which is hardly obtained in multilayers in visible/IR range, where a combination of both magneto-optical ferromagnetic layers and plasmonic metal films is needed^25^. On the other hand, compared to graphene, which allows magnetic tuning, these semiconductors have well-established manufacturing and are easy to work with in bulk form.

Different types of the SPR sensor architecture, discussed in this paper, offer significant adjustment of desired sensing properties. The material of the prism, thickness of the analyte, the angle of incidence and the magnetic field all provide wide parameter range for sensor design. The thickness of the dielectric (tens of micrometers) in the Otto configuration also points to applications with microfluidics, which operate with similar dimensions. Choosing another magneto-optical configuration (polar, longitudinal) which proposes controllable rotation and ellipticity can provide sensitivity to chiral molecules. This work shows, that it is possible to bring the benefits of SPR to the Terahertz range, along with an enhanced adjustment or modulation through the external magnetic field.

## Methods

**Figure 8:**
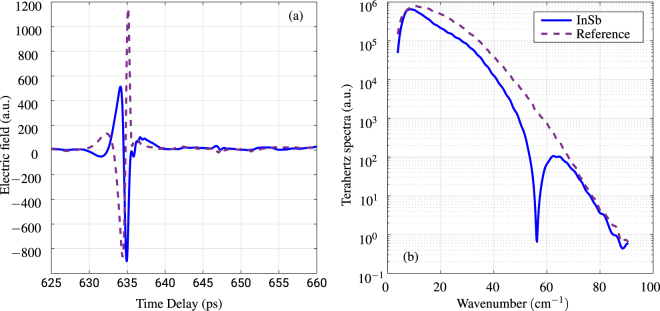
(**a**) Time domain signal of InSb SPR in Otto configuration and empty prism-reference. (**b**) Fourier Transform of the time domain signal.

We used the terahertz time-domain spectrometer (THz-TDS) Teraview TPS Spectra 3000, with the measuring range of 10–80 cm^−1^ in the ATR configuration. The principle of THz-TDS lies in excitation of a terahertz pulse from a photoconductive (PC) antenna using femtosecond (<100 fs) infrared (IR) laser pulse. The detection is performed via reverse process, where the terahertz pulse is sampled on PC antenna using the same IR laser, with variable time delay. Fourier transform is used to convert the detected time-domain Terahertz signal to frequency domain. The detected and transformed signal for reference (empty prism) and SPR on InSb are shown in Fig. 8. The subplot (a) is the time-domain signal, where it is possible to see a phase reversal due to reflection and surface plasmon coupling on the InSb. The subplot (b) is the transformed spectra. The ratio of the transformed spectra for InSb and Reference is the resulting reflectivity shown in Fig. 3(a). Blackmann-Harris three-term apodization^26^ is used to smooth the signal. The measuring chamber has been purged by nitrogen to avoid water vapor absorptions.

### Data availability

The datasets generated and analyzed during the current study are available from the corresponding author on reasonable request.
